# Why Are High-Achieving Students Susceptible to Inhibition? An Idiographic Analysis of Student Self-Identity in China

**DOI:** 10.3389/fpsyg.2019.01918

**Published:** 2019-08-21

**Authors:** Aruna Wu, Xiaowen Li, Jing Wang, Dan Li

**Affiliations:** ^1^Department of Psychology, Shanghai Normal University, Shanghai, China; ^2^Institution of Developmental and Educational Psychology, School of Psychology and Cognitive Science, East China Normal University, Shanghai, China; ^3^Minhang Teenagers Practice Education Base, Shanghai, China

**Keywords:** inhibition phenomenon of high-achieving students, self-identity, idiographic, emotional experience, semiotic mediation, sign

## Abstract

High-achieving students face greater expectations in competitive societies such as China, which can impede their performance. Based on previous observations regarding what we call the “inhibition phenomenon of high-achieving students,” wherein otherwise successful students show unexpectedly poor performances in collective activities of relatively unfamiliar forms, the present research analyzes the self-identity of such students and explores the underlying mechanisms that result in this inhibition phenomenon. An idiographic approach is employed to examine typical cases and their semiotic mediation in the self-identity regulative process. Two high-achieving students who exhibit the characteristics of the inhibition phenomenon are compared with another high-achieving student who appears not to be inhibited, using a multilevel and comprehensive analysis that integrates a number of aspects, such as the students’ emotional experience of the activities in relation to which the inhibition phenomenon occurs, their meaning-making regarding the activities, and their reflections on their daily school lives. The findings show that, for the inhibited students, a cued identity as being a “good student” is activated through the activities with the connotations of “being successful compared to the others” and “pursuing recognition” leading to a worsened performance; alternatively, the student not susceptible to inhibition displays an identity of being a “learner,” who focuses on the content of the activity and concrete suggestions from important others. These specific semiotic mediation processes indicate that, when self-identity is narrow and result oriented, it is easy for excessive future-oriented self-demands to be imposed, thereby bringing pressure to the individual at that moment. By contrast, a flexible and process-oriented identity facilitates an individual’s involvement in unfamiliar activities, enabling a richer, more open self-construction process.

## Introduction

Students with high academic achievement are usually recognized as great performers in school activities, yet, in our preliminary research, we found that some of these students perform poorly in particular types of tasks (e.g., Su, 2012; Zhang, 2018). Our prior studies, while having different aims, utilized similar methods, such as using playful activities like drama and storytelling, conducted in small groups of students with varying academic abilities. During these activities, which were separate from the daily teaching modules, students who normally attained high scores at school performed less well in these activities. They felt constrained and nervous and had low participation levels, and overall, their performance was poor. If other students with middle to low academic attainment were pro-active, the high-achieving students did not have the courage to try and had difficulty becoming involved in the activities. Although they were not necessarily expected to perform well in these activities, their strong emotional responses and conspicuously low involvement caught our attention. We identified these findings as the “inhibition phenomenon of high-achieving students” (hereafter, “IPHAS”), noting that it emerged in relation to collective tasks that were relatively unfamiliar in form to the student participants.

This phenomenon was first observed in a migrant school. “Migrant schools” are institutes especially established for the children of migrant workers. Almost all of their teachers and students come from the countryside but currently live in cities. Additionally, a recent study found that one of the characteristics of migrant schools is their non-comprehensive view toward education—that is, their emphasis on the development of arithmetic and literacy skills while disregarding learning through folklore, games, and activities (Wu et al., 2017). In fact, the students and teachers at the migrant schools are often familiar with such folklore, games, and cultural activities as they are from these traditional cultural backgrounds. Furthermore, it is likely that this singular focus on arithmetic and literacy skills in these schools influences how students perceive learning and achievement. Accordingly, the present paper analyzes IPHAS and reveals its uniqueness as well as its universality within these cultural and psychological facets.

The emergence of IPHAS is instigated by various interconnecting factors, including the age of the students and the society, culture, and history to which they are exposed. A student’s development typically will be full of unfamiliar tasks and fresh challenges. Consequently, if IPHAS persists, it could have a negative impact on the student’s mental development. Currently, academic success is an important indicator of students’ developmental success for most people in the context of contemporary China (Zhao et al., 2015). Close attention is paid to the physical and mental health of students with low achievement and poor performance scores in the situation, which gives prominence to academic achievement, and it is believed that students with high achievement scores have better development potential. However, the latter are also under a great deal of pressure. It is not hard to find in the media reports that high-achieving students’ mental health problems are becoming increasingly common. Because they generally exhibit good behavior, teachers and parents tend to neglect this group of students’ mental health, but, once such problems arise, they often lead to situations that are more serious. Moreover, an achievement-focused culture encompasses the risk of fostering the development of questionable values, which is common in a competitive society, as well as the neglect of its members’ physical and mental health (Damian et al., 2016). However, if this phenomenon is acknowledged and intervention is implemented in advance, latent problems can be minimized and the quality of these students’ lives can be improved.

There are different ways to understand IPHAS. Performance-oriented and ego-involved achievement goal/motivation theory (Nicholls, 1984; Dweck, 1986; Elliot and McGregor, 2001) suggests that students with high academic achievement have performance goals (i.e., to gain positive judgments or to avoid negative ones with respect to their competence in completing a task), and they are fixated on whether their ability can ensure affirmation from others. In addition, models of self-esteem and self-efficacy (Rosenberg, 1965; Bandura, 1982; Lane et al., 2004) suggest that low efficacy and self-esteem in related tasks in group activities will influence these students’ performances.

In this research, we analyze the phenomenon from the perspective of self-identity, as it is a more integrated and dynamic concept. First, “self-identity” integrates many mental functions and has a wide variety of implications. Put simply, “identity” means that the individual regards him-/herself as a certain kind of person (Gee, 2000). When this judgment is made, various mental functions may work together, including goals, values (Marcia et al., 1993), religious and spiritual beliefs (Gebelt and Leak, 2009), moral motivation (Hitlin, 2003; Hardy, 2006), motives, and self-assessment (Gregg et al., 2011). A high-achieving student’s performance on specific tasks involves an amalgamation of different processes, including a perception of the difficulty and characteristics of the task, an expectation of and reaction to other people’s behavior, an evaluation of their own ability from past experience, an estimation of the current performance, and an anticipation of what is about to happen. These processes are the results of the joint action of mental functions such as emotion, motivation, behavior, cognitive function, and reflective ability. Thus, if we only focus on one particular function, we cannot get a comprehensive and dynamic picture in authentic settings.

Second, identity integrates multiple factors such as history, culture, and social background. It is the term Erikson (1968) adopted, in his theory of identity development, to reflect what seems possible for oneself in a particular historical, cultural, and sociological time period. In a certain sense, then, identity may be conceived of as a social product and a cultural device, which can be more aptly expressed in terms of being the “funds” of identity (Esteban-Guitart and Moll, 2014). Identity exists and develops progressively and clearly in the constructive process balancing continuity and novelty, in the dialectic relationship between conformity and uniqueness, and in the continuous interaction with others. Undoubtedly, the causes underlying IPHAS are multiple, given the ways in which the social, historical, and cultural factors interact with individuals. Our study’s students are situated in, and reflect, their larger social and cultural backgrounds, which, in turn, are underpinned by the interactive processes of all these factors. Furthermore, the treatment of identity as an integrative concept facilitates further intervention by focusing on the factors related to sociocultural and historical backgrounds. Such an approach assists in fundamentally modulating the regulative processes of identity. Therefore, the analysis and exploration of identities within the social interactional framework can give rise to an intervention that is appropriate to its specific social settings. After all, a study’s participants are embedded in their social contexts, and this situatedness of each individual is taken into consideration. Through such deliberation, the resultant interventional plan can produce a more positive and long-term effect on the development of the study’s participants.

### Self-Identity as a Regulation Process Through Semiotic Mediation

Erikson’s (1968) proposal of and elaboration on the concept of self-identity inspired the present study. He posited that:

Ego identity, in its subjective aspect, is the awareness of the fact that there is a self-sameness and continuity to the ego’s synthesizing methods, the style of one’s individuality, and that this style coincides with the sameness and continuity of one’s meaning for significant others in the immediate community. (p. 50)

This suggests that identity is an integrated process of understanding and regulating the self, embodied in the maintenance of self-sameness and meaning in the immediate interaction with significant others. Based on Erikson’s theoretical formulation of identity, many researchers have trained their focus on the identity process taking place in the interaction between the individual and his/her particular surroundings (e.g., Adams and Marshall, 1996; Kroger, 2000; Bosma and Kunnen, 2001). Principally, an individual’s experience, emotion, and behavior in daily, specific events reflect the dynamic regulation process inherent in identity (Kerpelman et al., 1997). In recent years, researchers have also started to pay more attention to the dynamic regulation process of identity from a micro-dynamic perspective (Lichtwarck-Aschoff et al., 2008).

In this research, we will utilize the process-oriented concept of self-identity and analyze self-identity based on the framework of cultural psychology, which brings the symbolically regulated nature of mental activity to the forefront. Within this framework, psychological process is regulated through semiotic mediation (Valsiner, 2001), through which a person perceives and attributes values and meanings to their experiences (Nogueira, 2014). “Signs” are pivotal in the semiotic mediation process; they link the individual with the environment in a constant interaction that moves toward the future (Valsiner, 1999, 2001). Signs represent objects and yet take on new forms and meanings specific to the interpreter beyond the objects represented. Furthermore, signs do not occur in isolation; rather, they are co-constructed and constrained within social interactions. Generalization is one of the characteristics of the sign to create an abstracted reflection upon the initial context (Valsiner, 2001). Once transformed into a generalized and trans-situational form, signs can be integrated into the field of personal sense, from which they can be drawn upon and used in sense making under new circumstances, a process defined as “contextualization” (Valsiner, 2001). “Internalization” and “externalization” are the bidirectional construction processes that actualize the generalizing and contextualizing functions. The former transforms the “incoming” messages into a new form, while the latter composes these into new messages as the “output” for the social world to experience (Lawrence and Valsiner, 2003).

Based on this theoretical background, “self-identity” is further defined as hypergeneralized personal sense that functions as an implicit and unexpressed background that regulates sense making in our daily lives (Märtsin, 2010). Human sense making is regulated through signs that are hierarchically structured (Valsiner, 2001). In the hierarchical structure of a personal sign mediation system, self-identity corresponds with the signs on the highest level, overgeneralized, and speechless, while also deeply embedded into our functioning and sense making, powerfully guiding mental reactions in specific contexts. Generalization and contextualization are the reciprocal processes enabling the construction and reconstruction of self-identity, as well as being the regulative functions of self-identity. In specific activities, if internal reflective dialogues occur within an individual, it means that distinct regulative activities of self-identity are set in motion. According to Bakhtin Circle, the meaning and sense production process are social and ideological, dialogical, and multi-voiced. The meaning of an experience is produced in relation to multiple voices, internalizing different social positions and meanings (Nogueira, 2014). Signs within a regulative process are always future oriented, both in their immediate impact (turning the next immediate future into a new present) and in their general orientation toward encountering similar situations in indeterminate future moments (Valsiner, 2014). Thus, the dialogical process of the subject is reflected in past experience and future expectations entering the “here-now-I” system (Valsiner, 2002), which maintains internal stability in the dynamic process of interaction with the situation in the moment.

The identity process can be explained by social relations with social others and by a temporal connection linking the past, present, and future, both of which dialogically affect and constitute subjectivity. Therefore, in analyzing self-identity, we need to pay attention to the internal dialogue process and bring self-identity from the background to the foreground. Identity is distributed among persons, artifacts, activities, and settings and facilitates reflection over the emotional and cognitive processes of self-defining (Poole, 2017). Thus, in the context of the present study, it would be beneficial to explore the students’ identity in the activities where identity is embedded. From those specific activities, we expect to be able to perceive the actual regulation process of the abstract concept of self-identity, especially in relation to those activities that could arouse strong emotions.

In order to analyze the specific regulation process of identity, we will use the concept of “emotional experience,” as it can explain the uniqueness of the study participant’s understanding of and reaction to the situation, and it is a concrete manifestation of self-identity regulation. Emotional experience, based on Vygotsky’s “*perezhivanie*,” is the result of what influences a person and how these situations are comprehended and interpreted by the person (Nogueira, 2014). According to Vygotsky, an emotional experience (*perezhivanie*) is “a unit in an indivisible state representing the environment, i.e., that which is being experienced, as well as the ways in which the person experience, i.e., the personal characteristics and the environmental characteristics represented in an emotional experience” (Vygotsky, 1994, p.342). Thus, the emotional experience is a prism through which the influence of the environment on a child is refracted and through which we can see how a child becomes aware of, interprets, and emotionally relates to a certain event. It determines the role and the influence of the environment on the development of the child’s character and his/her psychological development (Vygotsky, 1994), and thus, the emotional experience can function as an analytic unit in a constant dialectic relationship between the representation of the outside world and how the world is experienced by the person.

### Idiographic Approach

In the present research, we conduct a case study of typical students with IPHAS through the framework of an idiographic approach. “Idiographic” approaches correspond to “nomothetic” approaches, with the former focusing on the particular and the latter on the general. The two are not antithetical or incompatible, however, but mutually inclusive and together strive toward generalized knowledge (Salvatore and Valsiner, 2010). Between the two approaches, idiographic research is often criticized for being inadequate in obtaining generalized knowledge, but, in fact, its goal is to pursue nomothetic knowledge through the singularity of the psychological and social phenomena. The criticism is often due to the fact that assumptions regarding research objects and mental activities are distinctly different between idiographic and nomothetic approaches.

The “ergodic” theorem is the basis for the nomothetic approach to sampling, with interindividual variability being the focus of psychological research (Molenaar, 2004); thus, it regards a specimen as homogeneous and population centered (De Luca Picione, 2015). Yet, psychological phenomena are non-ergodic and have their own idiosyncrasies. Idiographic approach conceives of mental activities as a self-organizing open system (Thelen and Smith, 2006). The occurrence of psychological phenomena does not follow simple causal relationships, but, rather, connections of relationships in a complex system. To build a local model of particular, contingent phenomena, researchers need to use abductive generalization to find general rules in complex phenomena.

Different from inductive generalization in the nomothetic approach, the idiographic process is conducted through abductive logic, which is aimed at promoting the creation of new knowledge through the generalization of the model of functioning of the single case (De Luca Picione, 2015). In the abductive logic, theory and evidence are circularly bonded within an open-ended cycle. Unlike the inductive inference, abduction does not pursue the general rule (namely, the regularities through the generalization of redundancies); rather, it uses the general rule (i.e., the background system of knowledge) in order to interpret the occurrences by reconstructing the phenomenon through which the occurrences acquire meaning (Salvatore, 2014). Thus, the idiographic approach is fundamentally open to variability, and it pays attention to deviations from the norm, transformation of phenomena over time, and dynamic and systemic aspects of phenomena (De Luca Picione, 2015).

Based on the above conceptual frameworks, this study examines IPHAS. In particular, we analyze typical students susceptible to the inhibition phenomenon and explore the causes underlying this phenomenon. Moreover, the study further establishes a theory that better explains the phenomenon.

### The Current Study

This research, focusing on IPHAS, analyzes high-achieving students’ emotional experience in the emergence of the inhibition phenomenon and discusses the self-identity process underlying such a phenomenon. The current study also constitutes a departure from further interventional research for students exhibiting the inhibition phenomenon. In future work based on the current research, the ultimate goal is to enable these students to achieve higher accomplishment in schoolwork as well as better mental health and overall wellbeing.

As noted above, the research adopts an idiographic approach. In doing so, it focuses on contingent and unique individual systematic cases and their subjective experience in order to build general understanding from a single phenomenon. In the research, inhibited high-achieving students’ emotional experiences, as well as their meaning making of their daily lives and important relationships, are analyzed and compared with those of other students who are not susceptible to inhibition. The in-depth discussion on IPHAS is approached through multiple perspectives—from micro to macro, from short to long periods of time, and from the participants to their interactions with culture and society. Specifically, two research questions will be explored in the research:

1. Why does IPHAS occur among high-achieving students who are susceptible to inhibition? What are their emotional experiences when IPHAS is present?
2. What are the characteristics of the self-identity of high-achieving students who are susceptible to this inhibition phenomenon?

## Materials and Methods

### Participants and Procedure

In this research, we focused on an activity in which inhibitive behaviors would be present to a greater degree. The activity centered on telling stories based on pictures provided. Four images were used from prior research (Wu et al., 2017), with these pictures respectively showing (1) students in a class, (2) two kids playing games, (3) a child talking with an adult, and (4) five children standing in a circle. These particular images were chosen because their themes would be familiar to the students and so would likely elicit stories more easily.

Three students formed one group; one was a student with high scores, while the other two students had achieved medium and low grades, respectively. The high-achieving student was selected based on their scores from the latest mid-term and final exams, with their average score ranking within the top 10% of their class (i.e., in a class of 60 students, they were among the highest-achieving six pupils); the other two students were ranked below the 60% measure. All three students in each group were asked to raise their hands to tell a story based on each of the pictures. When no one raised his/her hand, the picture was swapped for another one. The instruction given was as follows: “We will play a game. Please tell stories about the four pictures shown. There is no right or wrong, no good or bad about the stories. Just tell the story.” After the story-telling activity, students were interviewed, and they were asked to do a sentence completion test.

In this activity, in total, 22 groups of students were tested, with each group consisting of one high-achieving student and two students whose performances ranged from medium to low grades. This way of grouping gave us 22 high-achieving focal students in total. We measured the performance of the students by considering two factors: (1) reaction time and (2) the quality of story content. For the former, we defined students’ reaction time as the interval between the presentation of each picture and the moment a participant responded to the picture by raising their hand. For the latter, story content was evaluated based on the structure of the story with reference to a story-telling scoring system (Teglasi, 2001). After the analysis of the data, we derived four categories of high-achieving students based on the length of reaction time and the degree of the structure of story:

1. category A—students with longer reaction times and lower story structure scores than the average scores of the other two students in their group (12 students).
2. category B—students with longer or the same reaction times as the average score of the other two students, but whose story structure scores equaled or were higher than the average scores of the other two students (6 students).
3. category C—students with shorter reaction times and lower story structure scores than the average scores of the other two students (1 student).
4. category D—students with shorter reaction times and higher or equal story structure scores to the average scores of the other two students (3 students).

Herein, category A students’ performance was inhibited, while category D students performed well.

To gain an in-depth understanding of the characteristics of these kinds of students, the two most typical students from category A were chosen to be the focus of the study. These two students had been ranked among the top three students in their own classes, and they were widely recognized as “good students” by their teachers and classmates. Yet, in our designed story-telling activities, their performances showed a sharp contrast to their high academic performances. They felt nervous and found it difficult to be involved in the activities. In the following sections, the discussion is organized around these two students: “K” (male, 11 years old) and “J” (female, 11 years old). K was the class deputy in charge of studies, and J was the class monitor. They were from different classes and were both ranked at the top of their respective classes in the recent midterm and final exams. To analyze their uniqueness, the best performer (positive and active without retreat) among the three students in the category D grouping was used as a comparison: “L” (male, 11 years old) was the junior captain of his class and had an average score of the latest two exams ranking him sixth in the class. This study was carried out in accordance with the recommendations of the Guidelines of Academic Ethics, Shanghai Normal University. The protocol was approved by the Academic Ethics Committee of Shanghai Normal University. Written informed consent was obtained from the parents/legal guardians of all participants.

#### Video Data

The activity was recorded with permission from the teachers, parents, and students. It lasted about 60 min, with 30 min for each group.

#### Interview Data

Semi-structured interviews were conducted by two researchers following completion of the activity. In order to obtain as much comprehensive information as possible about the students’ responses, one of the researchers asked questions for the most part, the other acted as an observer, and both researchers recorded the students’ reactions for later analysis. The interviews consisted of two sections: the first focused on the individual experiences in the activity, while the second was an exploratory section. The interview in the first section included questions such as “Was the activity is interesting?,” “Which part was the most intense?,” “How did you feel then?,” “What were you feeling while others were telling the story?,” and “What was your self-demand in the activity?” The interview in this section aimed to gain an insight into the students’ behavior in the specific context, in order to better grasp the underlying meanings or their emotional experience.

The second, exploratory section was divided into three subsections. The first subsection pertained to daily life; it was aimed at understanding participants’ meaning-making process in relation to their daily lives. Questions in this part included reference to the happiest (and the most unhappy) thing about school and their views on the examination. The second subsection focused on the students’ self-images in their close networks; specifically, how parents, teachers, classmates, and friends viewed them. Self-evaluation and reflection come into existence when individuals establish relations and carry out interactions with others. Thus, this part of interview aimed to understand self-definition from different perspectives.

The third part investigated what kind of person they wanted to (and not to) be the most. The intention here was to explore their future-oriented possible selves, desirable and undesirable (Markus and Nurius, 1986).

During the interviews, based on students’ responses, and in addition to the above-noted questions, we also asked further detailed questions to elicit more nuanced information from the students. Each student was interviewed for about 15 min, and the interviews were audio-recorded.

#### Sentence Completion Test

Because students’ ability to self-reflect and to express themselves was still at a developmental phase, the extent to which the interview data could reflect students’ meaning construction and preferences was relatively limited. To make up for such a limitation of the interview data, we conducted sentence completion tests. The test was designed to better understand the students’ meaning making in situations inducing an inhibitory reaction, because, during the interviews and participant observations, we found that students who were prone to inhibitory responses felt nervous and held back. In the sentence completion test, three questions were featured, as follows:

1. Xiao Ming stood up and answered the teacher’s question, but the teacher did not give feedback. Xiao Ming ________.
2. Xiao Ming spent a lot of time trying to solve a very difficult question, but he failed. Xiao Ming ________.
3. Xiao Ming raised his hand to answer a question but gave the wrong answer. Xiao Ming ________.

The sentence completion test was conducted in the form of dialogue. Specifically, the interviewer uttered the first half of the sentence, which describes a situation, and the participants were then asked to finish the sentence as quickly as they could. This part was tape-recorded as well.

### Data Analysis

The data analysis comprised of the following four steps.

#### Step 1: Analysis of Video Data

Following the aim of the research, we adapted analytical methods from Jordan and Henderson (1995) and Heath et al. (2010). Analytic foci were selected by identifying fragments of video with meaningful events. In this study, according to the activity design and participants’ reactions, a story-telling activity triggered by one picture presentation was chosen as the key section, which had a clear start and end and which constituted a relatively independent section within the whole activity.

#### Step 2: Analysis of Interview Data

The data included two parts. The first part was participants’ interpretation of the activity, in which the participants could explain what they thought and felt at that moment. The second part was a general discussion of daily school life. Following the four steps of thematic analysis—coding, searching for themes, reviewing themes, and defining and naming themes—we identified themes of participants’ meaning making of daily life, important relations, and their selves (Braun and Clarke, 2006).

#### Step 3: Analysis of Sentence Completion Test

As complementary data to the interviews, the sentence completion test offered us information regarding the students’ understanding of the specific phenomenon and their unique experiences and perspectives in related situations instantaneously. Thus, the analysis in this step was carried out as a follow-up to the analysis of the interview and the projection test to understand students’ perception of ambiguous interactive situations and situations that lead to negative results. The sentence completion test was also used as supplementary material to gain an insight into how participants construct meaning.

#### Step 4: Summary and Integration of the Findings

In this last step, we made connections intra-individually and integrated at an idiographic level to understand and explain individuals’ emotional experience and dialogical process and to abstract participants’ self-identities.

Three researchers were involved in above processes of analyses. They watched the videos, discussed them with one another, and arrived at the research results that are presented in the next section.

## Findings

### Performance and Experience in the Activity

#### Performance in the Activity

The task of making up stories about pictures was unfamiliar to all students, but both J and K had top scores in their classes and their Chinese linguistic abilities—including expression ability—were recognized by their teachers, so the task should not have been difficult for them. However, when we look at the whole activity, these two students did not perform well. Four pictures were shown in turn. J only raised her hand for the last two pictures, and K only told a story about the last picture. Their stories were simple, comprising one to three sentences. By contrast, students with average academic performance told one to two stories about each picture, with every story containing more than two sentences. There was also some common behavior between the two students when each picture was shown, as follows:

1. In the first round, they were mainly excited and involved. When they saw the first picture, they showed a high degree of arousal, looked serious, frowned, and sometimes bit their lips and pens. When the others raised their hands, they told stories to themselves in a low voice with more concentration but never raised their hands. When the experimenter asked if anyone wanted to tell a story, they did not answer but bit their lips and fingers more.
2. In the second round, they were mainly conflicted and nervous. In contrast to their state of involvement in the first round, the two participants were not as focused and observed the facial expression of their peers and the experimenter frequently. When others raised their hands several times, both of them showed anxiety and regret. K moaned with annoyance, “Zi…,” while J lowered her head and picked at her fingers. When the experimenter asked if anyone wanted to answer, they both looked at the experimenter and looked like they wanted to but were afraid to respond. The experimenter encouraged them, “There is no right or wrong, no good or bad. You can raise your hand when the story comes into your mind.” But, they still did not raise their hands.
3. In the third round, there was either a breakthrough or a state of helplessness. When the third picture was shown, their anxiety became more obvious. J bit her lips all the time and blushed. K wiggled back and forth in his seat. When others raised their hands, K said, “Why are you [doing that] again? How can you still have [stories to tell]?” J looked at the experimenter after other two classmates finished. The experimenter asked her, “Do you have a story in mind?” She raised her hand and told a story: “They are singing hand in hand. Then it begins to rain heavily, and they all leave.” After that, she looked relatively relaxed. K still did not raise his hand once during the whole section. When experimenter gave a sign of changing the picture, K bent over and buried his face in his hands.
4. In the fourth round, they became involved and competitive. When the last picture was shown, their involvement improved. K changed his posture from leaning back against the seat to leaning forward and looking at the picture. When another classmate told a story, he started to prepare. He kept observing the experimenter, raised his elbow, and seemed to want to raise his hand but still hesitated. Finally, the experimenter asked K directly, “Do you have a story? It doesn’t matter if it is right or wrong. Just tell us.” He started to tell the story. Because of the experience in the third round, J was not as hesitant as K was, and she raised her hand to tell a story after other classmates finished.

By comparison, L was the second to raise his hand in the group when the first two pictures were presented, and the first to raise a hand when the third and fourth pictures were presented. The four stories had an average length of 14.5 sentences, all showing high levels of completeness in structure with a beginning, a climax, and an ending. The characters were depicted with personality and characteristics, and the content of the story had certain meanings. Let us take the third picture as an example. After seeing the picture, L thought for about 40 s before raising his hand, and replied:

Xuanxuan and Mingming are a pair of brothers. One day, the two brothers got up and looked for food. At this time, Xuanxuan broke his mother’s most beloved bottle, and immediately fled the scene and pushed it to Mingming. When their mother came back, she found that there were glass pieces that she liked on the ground, and asked them who did it. Xuanxuan said it was Mingming, and Mingming didn’t dare to say anything. He went out in the sunset to think about who exactly was wrong. Then he thought that, being the older brother, he should take on the responsibility, so he went back and told his mother that he did it. The mother beat him (Mingming) [and] Xuanxuan saw it and thought Mingming was very pitiful. He then took the initiative to admit the mistakes, and they became good brothers again.

In the first round of storytelling activity, L also started with excitement, and soon was involved in the process of creating a story, staring at the picture with his hand holding his chin. When another student next to him first raised their hand, he made no obvious movements, but, after the classmate finished speaking, he raised his hand to tell the story. The second story was created in a similar manner to the first. For the third picture, L was the first to raise his hand and told the story we have quoted above. After the fourth picture was presented, L reacted in a shorter time than for the third one and raised his hand high, unlike in the previous three rounds, when his hand was placed on the table. He was notably more excited and dedicated.

Examining J and K’s performance throughout the four rounds of the activity—from curiosity and involvement at the start, to tension and nervousness, to a state of either helplessness or breakthrough, and finally to involvement once again—their emotions are plummeting from excitement to inner tension, with anxiety mounting all the way through to the peak before the moment of raising their hands, after which the anxiety immediately abates. By contrast, L’s state of excitement maintained at a high level throughout the four rounds, even heightening before the end. He stayed highly involved during the process, and the story quality was relatively high.

#### Meaning Making of the Particular Events

We combined analysis of the students’ behavior in the activity with that of their feelings throughout the process and found that they had some common characteristics, as follows.

First, the activity was regarded as a learning or examination task. Even though we told them “we’ll play a game” to prevent them from being too nervous at the beginning of the activity, they still tended to treat it as an exam or a learning activity.

Researcher (R): Do you think this game is interesting?
K/J/L: [Yes, it is] interesting.
R: Why do you think it is interesting?
K: Our study can be improved through an exam like it.
/J: Because there is much knowledge we can learn.
/L: I have not seen this kind of picture before.
*Note:* “/” denotes answers to the same question from different students (here and below).

For students susceptible to inhibition, the fun of the activity was reflected in whether they could learn from it. If it was helpful in gaining knowledge or in improving their scores, it was an interesting activity.

Second, their performance in the activity was inferred to be equivalent to their intelligence. From their perspective, their performance in an exam depends on their intelligence; specifically, poor performance is due to not being smart enough.

R: Which section in this game did you feel most nervous?
J: Making up a story.
/K: Thinking about what to tell.
R: What are you thinking when you feel nervous?
J: I think I did not do well. I do not usually have a quick mind.
/K: He can make up [the story], why cannot I? … It seems that he is smarter than me.

L’s answer was somewhat different. He thought that the most stressful part was “the part where the little boy is squatting” (the second picture), the reason being that he was “worried about being wrong, about not being able to articulate the development of the story.” Furthermore, when asked “what if you don’t speak well?,” L said he was “afraid of [the] teacher’s criticism” and “of being criticized that I’m not concentrating enough.”

Their abilities are here being defined through comparison. Because the story-telling game was being compared to an intelligence competition, they set demands on themselves to show more intelligence, defined on the basis of comparison with others. For this reason, they set a self-standard of being as good as, or not worse than, others.

R: What do you worry the most in the game?
J: I have no idea. I’m afraid that they’ll think I’m stupid.
/K: When others answer the question but I do not, I think I have to work it out or I’m going to lose face.
/L: I’m worried that the teacher would criticize me for me not saying it well.
R: What are your expectations of yourself in the activity?
J: Be about as good as others, not the worst.
/K: Think and answer positively.
/L: Be serious. Do not fidget.

The students susceptible to inhibition have different responses when asked to choose the difficulty of the next task, but for almost the same reason—choosing the task that could protect their pride the most, whereas students not susceptible to inhibition used their own progress as their criteria.

R: If we played a similar game next time, what kind of picture will you choose: a harder, easier, or similar one?
J: Harder one, so that nobody can finish, and I will not be that nervous.
/K: Similar one. My Chinese is OK, but my imagination is not good.
/L: It can be harder, so I can progress faster.

In short, according to their performance in the activity and their reflection on it, students susceptible to inhibition believe that only activities helpful to their studies are beneficial, as they place a high value on study. They think that their differences in performance in the activity indicate differences in academic performance, which in turn indicates differences in intelligence. Intelligence as they mentioned is not absolute and defined intra-individually; on the contrary, it is a relative evaluation formed in comparison with other students. Alternatively, students not susceptible to inhibition focus on the content of the activity, and their self-evaluation is also mainly based on previous feedback from their teachers rather than in comparison with others.

### Meaning Making of Daily Life and Relationships

This section analyzes the data from the interviews and the sentence completion tests and encompasses three aspects: (1) the happiest (and the most unhappy) thing in the school and the student’s view on the examination; (2) the self in important relationships; and (3) the person they want to (and want not to) be the most.

#### The Happiest and the Most Unhappy Things at School Are About Examinations

Learning is the main activity in school. For the two inhibited students, the event that triggered the strongest emotional responses was related to the result of the learning activity—the examination.

R: What is the happiest thing in school?
J: When I win the exam.
/K: When I get higher score than others.
/L: When the teacher praises me.
R: What is the unhappiest thing in the school?
J: Being terrible on my test.
/K: When I fail my examination.
R: Why? What do you worry about?
J: Disappointing my parents.
/K: My parents would criticize me.
. . .
R: What if they criticize you?
K: They will be sad.
. . .
R: What do you worry about the most?
/L: [That] my teacher would criticize me, like, really fiercely.

They valued success in the examinations the most, as measured by achieving higher scores than those of other classmates. Meanwhile, the most unhappy thing was doing poorly on examinations, because they do not want to let their parents down. The most happy and unhappy things for L are related to the teacher, as the teacher’s evaluations are very important to him. At the same time, the reason for his worry about the teacher’s criticism is that “the teacher is particularly fierce.” This kind of worry is related to the teacher’s attitude, rather than L’s own behavior potentially disappointing the teacher.

#### A Good Relationship Means Being Recognized for High Academic Performance

This section refers to the students’ relations with teachers, parents, and peers. Being recognized and being mocked or even criticized based solely on academic performance was a universal theme reflected in the students’ perception of others’ opinions toward themselves in the interview.

R: What kind of kid does your father think you are?
J: Lazy. He said that I am smart and am able to learn better, but just lazy.
/K: Just a common student who does not work hard.
/L: Careless. Sometimes, when you do math, you are always wrong, when it could have been right. When I solve an applied math question, sometimes I do it without looking at the question first.
R: What kind of child does your mother think you are?
J: Conceited and easy to be proud. My mother is always dissatisfied with me.
/K: My mother thinks I’m great. Always getting high scores.
/L: Always thinking about playing.
R: What do your teachers think?
J: Proud of me. On Math, although I did arithmetic slowly, the accuracy is pretty good.
/K: Good student. They let others learn from me.
/L: Quite loveable, just that the voice is a bit small.

The interview data suggest that these students can get teachers’ praise as long as they perform well in school, but their parents have higher standards for them at home. The responses regarding their parents’ idea of them are almost all about scores. It may be common to talk about scores in parent-child dialogues. L’s performance at home and at school is also related to learning. This is a reflection of the overall social atmosphere of the study’s setting. Everyone attaches great importance to learning. However, the evaluations from Dad and the teacher mentioned by L are relatively specific, including carelessness on a certain type of math problem and the small voice.

There is no particular tendency about the self in the eyes of friends in the interview data, but the results of the sentence completion tests suggest that they are worried about mockery from their peers.

R: Xiao Ming raised his hand to answer the question, but he said a wrong answer. Xiao Ming ________.
J: Xiao Ming thinks he is the worst.
/K: He will sit down awkwardly and lower his head. Because his classmates look at him with a mocking look, he will feel very unpleasant.
/L: Xiao Ming is not discouraged, and asks the teacher what the answer is.
R: Xiao Ming spent lots of time trying to solve a very difficult question, but he failed. Xiao Ming ________.
J: Why cannot I solve the problem? Even Xiao Song can do it. They must think I’m quite stupid.
/L: Xiao Ming continues to think, yet still cannot think of an answer. If it does not work, he would ask the teacher after class.
R: Xiao Ming stood up and answered teacher’s question, but the teacher did not give feedback. Xiao Ming ________.
K: He feels so sad, the teacher seems to look down upon him.
/L: Xiao Ming feels very sad because the teacher ignored him.

When they face some challenging tasks or events with negative results, they first think about others’ opinions on them. Good performance means getting others’ recognition, while bad performance means a setback for the relationship with important others. For example, the teacher would look down on them, and peers would mock them. It also shows that their relationships are attached to scores, not to other aspects in the relationships. L thinks more about the problem itself, and, if he cannot solve it, he will ask for help. He does not feel that these things will bring harm to his self-esteem. But, at the same time, it can be seen that he is more concerned about the teacher’s evaluation and affinity toward him.

#### The Desirable Self Is a Smart or Popular Person

The idea of the type of person one wants to be or not to be is similar to the notion of possible selves put forward by Markus and Nurius (1986). It focuses on the desirable and undesirable self, which together provide an evaluative and interpretive context for the current self. The two high-achieving students’ answers to this question show where they pay the most attention.

R: What kind of person do you want to be the most?
K: Smart.
R: What is smart like?
K: The person who can do everything.
R: How far are you away from that?
K: I’m not good enough on math.
R: What kind of person do not you want to be most?
K: The most stupid.
R: What is that like?
K: The person who can do nothing and has the lowest score.

Smartness is the goal that K wanted to achieve the most, as manifested in academic achievement. He thought he was not good enough on math, which means he was not satisfied with his math score.

R: What kind of person do you want to be most?
J: Active and [a] cute person liked by everyone.
R: What is an active and cute person like?
J: Liked by everyone.
R: How far are you away from that?
J: Not so far away, just a little more cuteness.
R: What kind of person do not you want to be most?
J: The arrogant one.
R: What is arrogant like?
J: The person who cannot do something as told by others many times.
R: Who are the others? Who will say it many times?
J: Teachers and also parents. But they still cannot do it well.

J wants to become the most popular student. Combined with the person she does not want to be the most, it can be seen that what she really wants to get is recognition from others in a relationship. If she cannot meet the standards of others, she cannot get affirmed, and thus, her self-worth is negatively affected.

The person L mostly wants to be “an artistic student with good scores in Chinese, math, and English, because in this way one would have a good future.” For now, he is “some distance away” from his goal. The person he does not want to be the most is “a person who does not finish his homework, and a person who does not write Chinese characters well.” L’s desirable self is also related to learning, but it does not show obvious characteristics of comparing oneself with others, but, rather, of individual performance in respect of specific things. Specificity and individuality are the two notable characteristics of his ideal.

### Generalized Identity: Signs in the Dialogical Process

#### The Interviewing Process of Past, Present, and Future

K and J were excited at the beginning of the storytelling, but, with others raising hands during the activity, they became nervous. This reflects the contradiction between self-expectation and real performance in the moment. Expected behavior pointing to the future is formed based on past experience. For these two students, their past positive experience is produced and consolidated by being awarded favorable feedback through learning activities; thus, they might regard certain activities as a test or learning activity, because this is the field where they do best and with which they are most familiar and have an expectation of good performance.

The other students’ behavior of raising their hands in the activity is interpreted by the focal two as the others having better performance than them. The high-achieving students here think they are not as “good” as the students with average or low scores. This system of interpretation shows the characteristics of their meaning construction. First, their criteria of evaluation stress linear performance but lack more diversified content. The standards of “good” and “bad” are defined by the order in which hands are being raised. Second, the dynamics of self-position are displayed: they believe that their performance in one activity will influence the whole self-position. Based on this system of meaning construction, J and K start to feel nervous and anxious because of their lackluster performance in the current activity. According to the processing efficiency theory (Eysenck and Calvo, 1992), anxiety has negative effects on emotionality. The individual is excessively aroused due to nervousness and anxiety, which occupy part of the cognitive resources, and this negatively affects the completion of tasks.

Meanwhile, the future-oriented anticipation in experiencing the present formed on the basis of past experience is to surpass the others (or not to lag behind them) and get others’ affirmation. Thus, the demands set for themselves are to make up better stories. They are afraid that their stories are not good enough, so they do not dare to raise their hands, which ultimately leads to inhibition in tasks in which they should have performed well.

L’s many self-evaluations are also related to learning, but the strong sense of evaluation of being either good or bad is not evoked. He is easily involved in the activity because he is attracted to the content and process of the activity itself. In his symbolic regulation, there is a weak process of abbreviation (Valsiner, 2014), which also leads to less constraint in his future-oriented action, creating more possibilities for his performance in the activity.

#### The Dialogue With Imagined Others

Identity is constructed in the interaction with significant others, which is reflected in two aspects for our study’s students. First, they define their abilities in comparison with others. A student is a good student only when he/she surpasses or does not lag behind others. As a result, they can be affirmed by teachers and parents for their performance, which further promotes the relation with their peers and the formation of their self-identity. Thus, only by regarding the game as a learning activity or as an examination, they can make comparisons and gain praise. At the same time, it is important that parents and teachers pay attention to their learning, which makes them tend to be involved in learning activities in order to get positive results—that is, high scores.

In the activity, J and K expressed their worries about others’ negative evaluation. They are very passionate about high scores and believe that, if they do not show a good performance, they’ll be ridiculed by their peers (e.g., “they will call me stupid if I can’t make up the story”). The others and their evaluation only exist in their imagined world. The evaluation is not from a specific person but a generalized group. Meanwhile, the tendency to compare with others has been generalized in school activities: even the happiest thing in school is getting better scores than others and getting an award. The generalized experience is formed in the imagination process without specific objects, and the emotional experience extends to different situations, which contributes to an individual’s specific identity.

By contrast, L also showed a dialogue with imagined others (mainly teachers) in the activity and the interviews. There are two aspects that concern him the most (have the most meaning) in terms of the teachers: (1) the specific guidance provided by the teachers—even when it is criticism, it is focused on specific actions such as lack of concentration and (2) the affinity with the teachers. For J and K, their evaluations of important others are either general (e.g., being praised or recognized) or ones related to self-esteem (e.g., being looked down upon), whereas, for L, it is the close connection with the teacher that he pays most attention to. He is worried about being criticized by the teacher not because he feels that he is not good enough, but because the teacher is very fierce. Therefore, in this mode of dialogue, his behavior has specific guidelines and requirements, is situational, and is more flexible. He will not be too anxious in this process.

#### The Identity of “a Good Student” Aroused in the Activity

**Figure 1:**
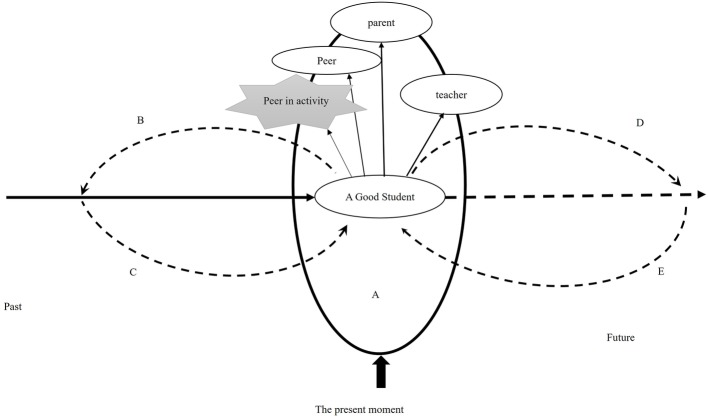
Self-identity process of the two inhibited students in the activity.

J and K do not want themselves to be those “bad” students who have low scores, are “stupid,” and not welcomed by classmates. By contrast, the standards set for themselves are to be “smart,” “popular” students who “have higher scores than others,” “are awarded,” and “don’t disappoint their parents.” Thus, J and K have the “good student” identity, a cued identity activated by the activities, the interviews, or even the school context. The dialogue process of this self-identity in the activity is presented in Figure 1.

As shown, on encountering a relatively unfamiliar activity, the individuals automatically retrace their histories and try to find references to understand the activity (Process B). It is natural that they interpret the current unfamiliar activity as learning or as an examination, because learning is their most important and confident activity (Process C). With the co-effect of B and C, the individual’s “good student” position is activated, leading them to make demands oriented to the future and consistent with the past, such as “surpassing others”/“not behind others” and “getting affirmation” (Process D). The orientation of the future anticipation to the current action functions on the present moment (E). But, the students’ perception of their peers’ good performance in the moment makes them feel that they are not as good as others (A), which contradicts with their future-oriented anticipation. Meanwhile, they need to deal with the evaluation from their imagined teachers, parents, and peers in the present self-meaning—system (A), where their “good student” position is disturbed, which engenders tension and withdrawal.

We continue to expand this model to the general activity. The students’ self-structure features strong “score-orientation.” The content of the self as a “good student” is narrow, and the positioning process operates through comparing themselves with their peers. They subconsciously compare themselves with others, judge themselves from others’ views, and worry about others’ negative comments. So, with the narrowness of score orientation, their self-demands become unidimensional and often too high to be attained by realistic action. Thus, the feeling of tension from not meeting self-demands threatens the original and the past self.

**Figure 2:**
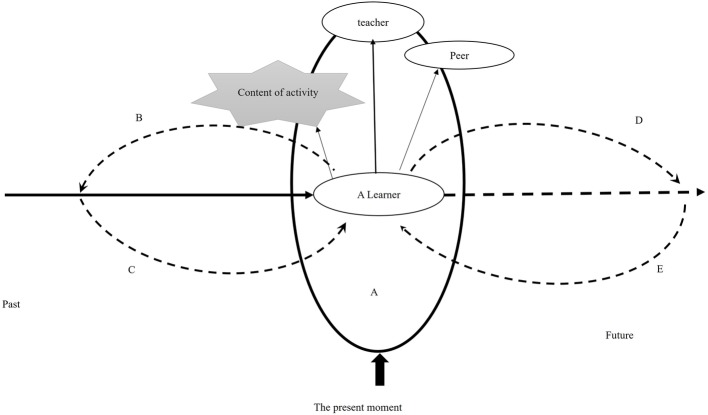
Self-identity process of the uninhibited student in the activity.

The contrasting identity process of L is illustrated in Figure 2. L is an active participant in the activity. He wants to learn and to gain some knowledge and experiences through the activity. Therefore, in the face of difficult and ambiguous situations, he will try to find the answer, to ask the teacher for help, and to ask a companion. He shows a “learner” position, reflected not only in the activity throughout which he was highly involved, but also during the reflection on the activity when he expressed that the pictures were very interesting as they had never been seen before. When recalling the most stressful part of the activity, he also mentioned specific picture content. Thus, the content of the activity constitutes important signs for him. The meaning extracted from the past experience (process B) is obtained in comparison with the current activity (process C). When he found that the activity content was novel, this meant fun and gain. In the meaning system at this moment, important others, especially teachers, are also activated. In the dialogue with the latter, what he seeks is emotional affinity and behavioral guidance, so he is also more likely to take appropriate action in line with the voice of significant others in the activity. This is different from the content of inhibited students’ internal dialogue system (seeking recognition generally). For all of these participants, the more contextual and specific the voice, the more possibilities the future-oriented expectations and actions (D,E) will have, and the less constrained the subject’s own standards are.

## Discussion and Conclusion

Our research compared two typical students who are susceptible to inhibition and one student who is not susceptible to inhibition in order to understand the self-identity of high-achieving students displaying what we have described as an inhibition phenomenon. The students susceptible to inhibition activated the identity of “good students” in the activities, looked back to personal history to find support, and made future-oriented demands for themselves. Conflict was present between the currently perceived performance and self-demand that motivated the students to succeed in comparison with others and to be affirmed. This conflict in turn stimulated their strong emotional responses and presented obstacles to their performances. Thus, the self-identity of these two students was found to be narrow and result-oriented; it was easy for them to impose excessive future-oriented self-demands, thus bringing pressure to themselves in the moment. Similar to the students who were susceptible to inhibition, the student who was not susceptible to inhibition was also very concerned about learning, and they also placed emphasis on evaluations from significant others in the internal dialogue, but the difference was that his concern was not about the score but the material content of learning; the evaluation from others was not overly abstract but concrete. Both the concern about learning and evaluation from others led to the identity of a “learner” that he activates. Thus, flexible and process-oriented identity facilitated his involvement in the activities, enabling a richer, more open self-construction process.

A highly generalized self-identity, as a background, determines which signs an individual capture in the specific activities in which they participate and defines the specific ways in which they understand the signs. Students susceptible to inhibition had an intense reaction to their “peers’ raising hands” during the activities, which translate as “good performance in the activity” for these academically high-achieving students. The high-achieving students’ excitement and involvement in an instant turned into stress and anxiety as other students raised their hands. This particular hand-raising sign has special meaning for these academically excelling students and led them to retreat back from activity, which clearly resulted in their low-involvement in the activity. “Why are you [doing that] again? How can you still have [stories to tell]?”: these quotes from K can best represent his state of mind. On the other hand, when J hesitates, the questions from the experimenter, including the experimenter’s prompts in the fourth round, act as a promoter sign (Valsiner, 2014) counteracting her resistance and facilitating her quitting the nervous state. The other significant sign is “scores”—the numerical signs indicating smart or stupid, excelling or falling behind, or, more directly, “good” or “bad.” High “scores” are equated with being a “good student,” and the difference between “good” and “bad” students simply means overtaking and falling behind others. The student not susceptible to inhibition, however, pays attention to the object of the activity; that is, the content of the picture. His “learner” identity requires him to pursue personal progress, which further drives him to be involved in the activity. The subsequent picture presentations also provide an opportunity for him to pursue his goal, acting as a catalyzer (Valsiner, 2014) to help him improve throughout the activities, until the fourth round, when he can quickly tell the story with excitement. In fact, this also shows that identity, as a high-level sign, transforms the individual’s environmental information into conditions that promote the development of the individual in the regulation process.

From the perspective of the regulation process of self-identity, the identity of “good student” of the students susceptible to inhibition coincides more with the concept of “ought self” proposed by Higgins (1996). They perceive the expectations from their parents, teachers, and classmates in the activities, and they feel anxious and stressed when those expectations differ greatly from the current state. The content of “ought self” is more about the expectations from important others for their academic achievement. Furthermore, formation of “ought self” is also related to the current general anxiety experienced by the whole society. Learning tends to be more utilitarian in China and other competitive societies. Academic success means a lot in this cultural context and encompasses respect from others, family pride, and social mobility (e.g., Kipnis, 2011). Thus, this atmosphere results in unidimensional values and evaluation systems. Individuals’ understanding of “good and not good” is unidimensional and deficient in content. In our study, the narrow meaning of these signs is due to students’ lack of participation in activities that put emphasis on self-exploration and diversity. Once these students encounter unfamiliar situations, their interpretation of such circumstances will greatly depend on their previous experience of learning. If the activities for reference are more diverse, they can then have more diverse self-demands, as opposed to the unidimensional study performance, and they can be more flexible in different activities. Also, they can protect themselves better and develop in more positive ways.

We interpret IPHAS in the frame of semiotic mediation, which provides clues for further intervention. The identity of “good student” may lead to confusion and anxiety, but it is also an opportunity for development; it provides these individuals with impetus for further development. Only when the meaning making of “good” deviates or narrows, it does cause problems. Thus, in future interventions, we can start from here—strengthening an individual’s “good student” identity and providing richer meanings to replace the original “comparing with others” and “getting affirmed,” channeling energy in the process of the individual becoming a true, value-diverse “good student” (Oyserman et al., 2002). Based on individuals’ agency, we can activate their inner force for a healthier and more positive development.

## Data Availability

All datasets generated for this study are included in the manuscript and/or the supplementary files.

## Ethics Statement

This study was carried out in accordance with the recommendations of the Guidelines of Academic Ethics, Shanghai Normal University. The protocol was approved by the Academic Ethics Committee of Shanghai Normal University. Written informed consent was obtained from the parents/legal guardians of all participants.

## Author Contributions

AW wrote the first draft of the manuscript and conducted study design, data collection, and data analyses under the supervision of XL and DL. XL gave the instruction and critical feedback on study design and data collection. DL instructed the process of data analyses and draft writing. JW assisted the data collection and revised and polished the manuscript.

### Conflict of Interest Statement

The authors declare that the research was conducted in the absence of any commercial or financial relationships that could be construed as a potential conflict of interest.
